# Epidemiology and treatment trends in cervical, thoracic and lumbar disc herniation– a nationwide analysis

**DOI:** 10.1016/j.bas.2026.107448

**Published:** 2026-09-18

**Authors:** Joan Alsolivany, Anton Früh, Ahmad Almahozi, Nora Dengler, Nils Hecht, Peter Vajkoczy, Christina Susanne Mark-Sargut, Tarik Alp Sargut

**Affiliations:** a Department of Neurosurgery, Charité – Universitätsmedizin Berlin, Charitéplatz 1, Berlin, 10117, Germany; b Department of Neurosurgery, HELIOS Hospital Bad Saarow, Pieskower Straße 33, Bad Saarow, 15526, Germany; c Department of Anesthesiology, Charité – Universitätsmedizin Berlin, Hindenburgdamm 30, Berlin, 12203, Germany

**Keywords:** Lumbar disc herniation, Cervical disc herniation, Thoracic disc herniation, Epidemiology, Nationwide analysis

## Abstract

**Objective:**

Degenerative disc herniations are a major contributor to neurological disability and healthcare utilization. However, nationwide data on temporal demographic trends and treatment patterns in cervical and thoracolumbar disc herniation remain limited. This study aimed to analyze long-term epidemiological and treatment trends in Germany over a 10-year period.

**Methods:**

This retrospective population-based study analyzed nationwide hospital discharge data from the German Federal Statistical Office between 2014 and 2023. Patients hospitalized with cervical disc herniation (CDH; ICD-10-GM M50.+) or thoracolumbar disc herniation (TLDH; M51.+) were included. Demographic characteristics, hospitalization rates, treatment procedures, comorbidities, and temporal trends were evaluated descriptively and stratified by diagnosis category.

**Results:**

A total of 1,495,546 inpatient cases were identified, including 1,254,674 TLDH cases (83.9%) and 240,872 CDH cases (16.1%). The most affected age group was 45–60 years, with a slight male predominance. Since 2018, a demographic shift toward older patients was observed, with the proportion of individuals aged ≥80 years increasing from 2.1% in 2014 to 4.4% in 2023. Hospitalization rates declined substantially after 2020. Mean length of stay decreased from 6.0 to 4.5 days for TLDH and from 7.1 to 5.2 days for CDH. Injection-based therapies declined markedly, whereas operative volumes remained comparatively stable. The most frequent comorbidities were hypertension, diabetes mellitus, and coronary artery disease across both cohorts throughout.

**Conclusion:**

Hospitalizations for CDH and TLDH declined over time, while the proportion of elderly patients increased considerably. The COVID-19 pandemic temporarily reduced hospitalization rates, and hospital stays shortened substantially, reflecting ongoing structural changes in spine care.

## Highlights

• Nationwide analysis of 1.5 million inpatient cases of cervical and thoracolumbar disc herniation in Germany between 2014 and 2023.
• Disc herniation predominantly affected the working-age population, while the proportion of patients aged ≥80 years doubled over the study period.
• Hospitalization rates declined substantially after 2020, reflecting a marked impact of the COVID-19 pandemic on spine care utilization.
• Injection-based spinal pain therapies decreased by 43% over the decade, whereas surgical treatment volumes remained comparatively stable.
• Mean hospital length of stay decreased by approximately 25%, indicating increasing efficiency in inpatient spine care.

## 1 Introduction

Cervical, thoracic, and lumbar disc herniation (CDH, TDH, LDH) are the most common causes of radicular pain and functional disability, particularly in the working-age population (Konstantinou and Dunn, 2008). Although most patients benefit from conservative therapy, including analgesia, physiotherapy, and structured pain management programs, surgical intervention remains indicated for persistent symptoms or neurological deficits (Weinstein et al., 2006; Atlas et al., 2005). Over the past decade, advances in minimally invasive spine surgery and strengthened guideline recommendations for non-operative management have driven changes in clinical practice (Evaniew et al., 2014; Surgery versus conservative management of sciatica due to a lumbar herniated disc: a systematic review et al., 2011; Jin et al., 2025).

Parallel to these developments, demographic shifts, an increasing burden of comorbidities such as obesity and metabolic disease, and the COVID-19 pandemic may influence the presentation and treatment decisions for patients with CDH, TDH, and LDH.7,8.

Despite their clinical and socioeconomic relevance, nationwide data on temporal trends in hospitalizations and patient characteristics for cervical, thoracic, and lumbar disc herniation in Germany remain limited (Katz, 2006; Andersson, 1999).

This study analyzes administrative data from a nationwide hospital registry in Germany since 2014 to evaluate trends in cervical, thoracic, and lumbar disc herniation over time, including the COVID-19 pandemic, focusing on hospitalization, the proportion of surgical versus conservative management, and changes in patient demographics and comorbidity profiles.

## 2 Methods

This retrospective, population-based study analyzed nationwide hospital discharge data provided by the Federal Statistical Office of Germany (Statistisches Bundesamt, Destatis).

The dataset comprises all inpatient cases treated in German hospitals and includes diagnostic and procedural coding according to the International Statistical Classification of Diseases and Related Health Problems, 10th Revision, German Modification (ICD-10-GM), as well as the German Operation and Procedure Classification System (OPS). Data were aggregated annually from 2014 to 2023. All records were fully anonymized and contained no patient-identifiable information; therefore, ethical approval and informed consent were not required.

Hospitalizations with a primary diagnosis of cervical, thoracic, or lumbar disc herniation were identified using ICD-10-GM codes M50.+ (cervical) and M51.+ (thoracolumbar). Owing to the ICD-10 coding structure available in the DESTATIS database, thoracic and lumbar disc herniations were analyzed jointly under the M51.+ category.

To restrict the cohort to degenerative etiologies, cases with concomitant diagnoses indicative of infection (M46.*, G06.*), neoplasm (C* or D*), or trauma (S*) were excluded. Patients were stratified according to spinal region (cervical and thoracolumbar), sex, and predefined age groups (<40, 40–60, 60–80, and >80 years).

Operative management was defined by the presence of relevant OPS procedure codes for lumbar laminotomy, sequestrectomy, microdiscectomy, or fixation (e.g., 5-030.+, 5-031, 5-832, 5-831.+, and 5-830.+), as well as ventral discectomy (5-831.2), ventral fixation (5-836.+), or discectomy via a dorsal cervical approach (5-030.+). As the DESTATIS database records all OPS-coded procedures performed during an inpatient stay, multiple diagnostic, interventional, and surgical procedures may be assigned to a single hospitalization. OPS data were available as aggregated procedure counts rather than patient-level treatment indicators. Consequently, procedure counts cannot be converted into the proportion of hospitalizations involving at least one surgical procedure, and no patient-level procedure denominator could be calculated from the available aggregated data.

Comorbidities were identified from ICD-10-GM secondary diagnoses recorded during the respective inpatient stay. As administrative coding follows German coding guidelines, only comorbidities considered clinically relevant for the hospitalization are routinely documented. Consequently, the reported frequencies should not be interpreted as prevalence estimates of chronic diseases within the study population.

Descriptive statistics were calculated for all variables. Temporal trends across the entire study period were assessed using simple linear regression with year as the independent variable. Regression coefficients (β), 95% confidence intervals (CI), coefficients of determination (R^2^), and p-values were reported. To further assess whether the onset of the COVID-19 pandemic was associated with a change in hospitalization trends beyond the pre-existing temporal trajectory, an additional segmented linear regression analysis was performed with a prespecified breakpoint at 2020. The model estimated the underlying pre-2020 temporal trend, an immediate level change in 2020, and a change in slope thereafter. The breakpoint was defined a priori based on the onset of the COVID-19 pandemic rather than identified in a data-driven manner. Model assumptions were assessed using residual diagnostics, including evaluation of serial autocorrelation and heteroscedasticity. Heteroscedasticity-robust (HC3) standard errors were additionally examined as a sensitivity analysis. Given the limited number of annual observations, estimates derived from the segmented regression were interpreted cautiously. Differences in age distribution between cervical and thoracolumbar disc herniation were analyzed using Pearson's chi-square test. The initial descriptive and simple regression analyses were performed using GraphPad Prism (version 10), while segmented regression and sensitivity analyses were performed using Python version 3.13.5.

A two-sided p-value <0.05 was considered statistically significant. Statistical analyses addressed prespecified epidemiological and treatment-related outcomes. Given the descriptive and exploratory nature of the study, no formal adjustment for multiple comparisons was applied; individual p-values should therefore be interpreted accordingly.

## 3 Results

Between 2014 and 2023, a total of 1,495,546 inpatient cases of cervical disc herniation (CDH) and thoracolumbar disc herniation (TLDH) (ICD-10-GM codes M50.+ and M51.+) were treated nationwide in Germany. Of these, 730,433 patients (48.8%) were female, indicating a nearly balanced sex distribution (Fig. 1.).

**Fig. 1:**
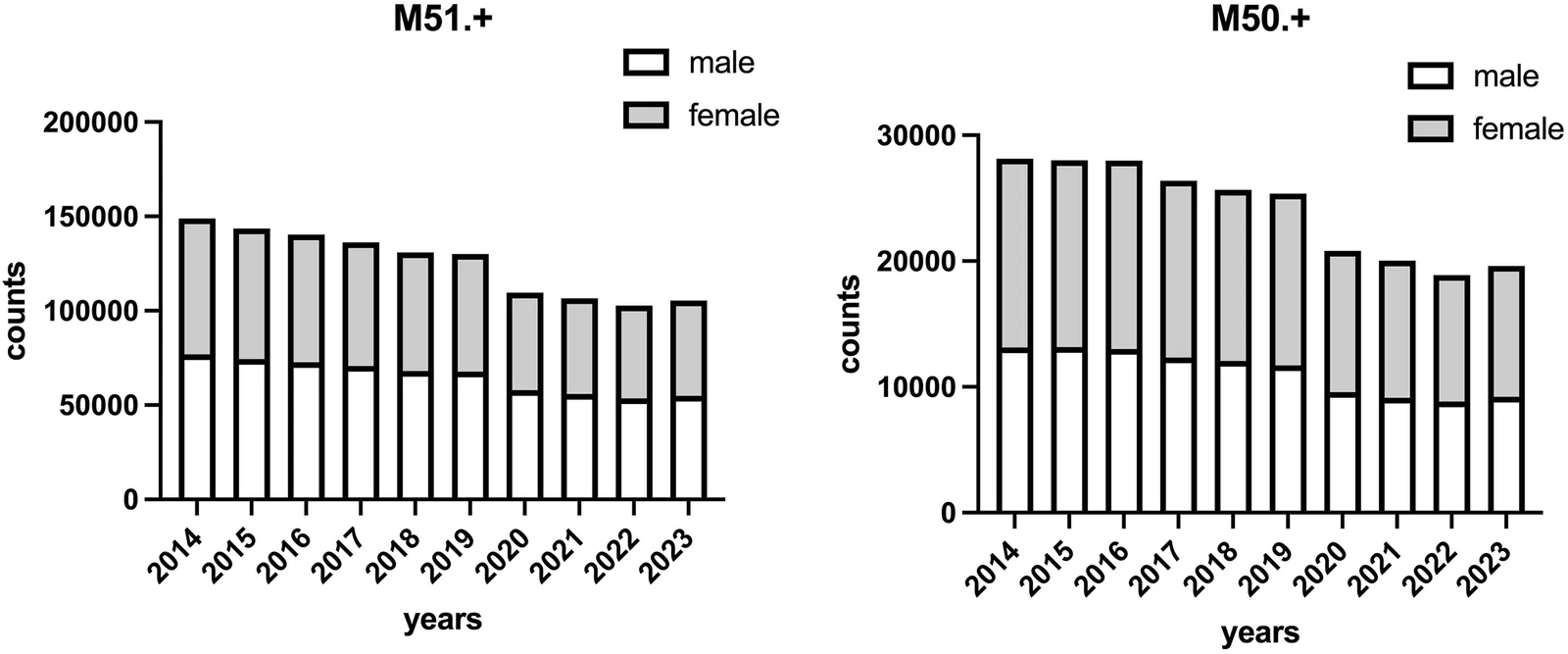
Annual inpatient hospitalizations for cervical (M50.+) and thoracolumbar (M51.+) disc herniation stratified by sex in Germany between 2014 and 2023.

Thoracolumbar disc herniation (M51.+) accounted for the majority of cases, comprising 1,254,674 patients (83.9%), whereas cervical disc herniation (M50.+) accounted for 16.1% of all hospitalizations. This proportional distribution remained stable throughout the observation period.

Over time, a substantial decline in hospitalization numbers was observed. TLDH cases decreased from 148,936 in 2014 to 105,469 in 2023, while CDH cases declined from 28,133 to 19,600. Hospital volumes remained relatively stable until 2019, followed by a marked decline beginning in 2020. Compared with 2019, admissions decreased by 16% in 2020, corresponding to an overall reduction of 26% compared with 2014. Although a partial recovery was observed after the peak pandemic years, hospitalization numbers did not return to pre-pandemic levels (Fig. 2).

**Fig. 2:**
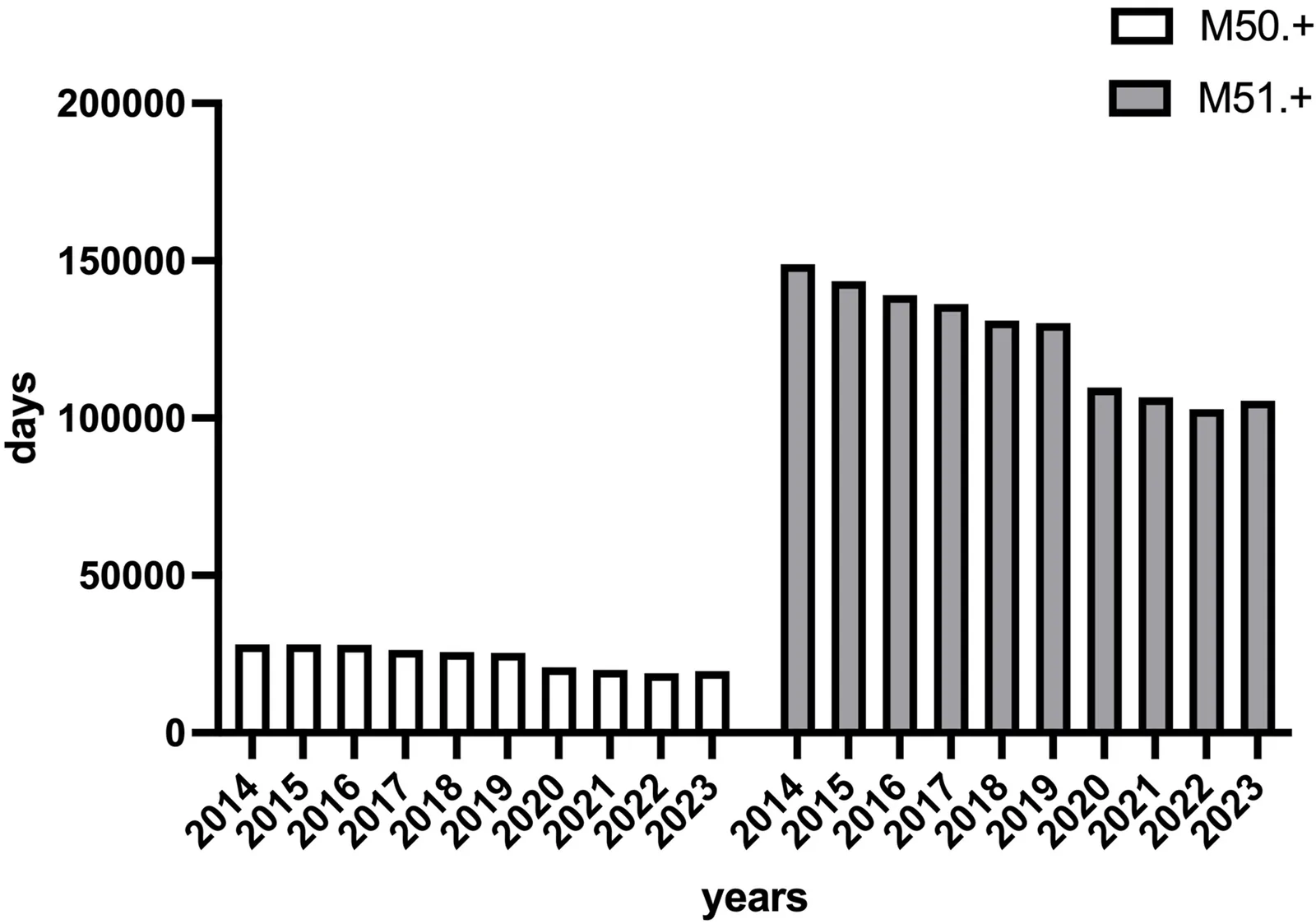
Annual inpatient hospitalizations for cervical (M50.+) and thoracolumbar (M51.+) disc herniation in Germany from 2014 to 2023.

Linear regression confirmed significant downward trends in both diagnostic groups. Hospitalizations for cervical disc herniation decreased by an average of 1197 cases per year (β = −1197 cases/year; 95% CI −1514 to −879; R^2^ = 0.904; p < 0.001), while thoracolumbar disc herniation declined by 5573 cases per year (β = −5573 cases/year; 95% CI −6807 to −4338; R^2^ = 0.931; p < 0.001).

In an additional segmented regression analysis with a prespecified breakpoint at 2020, hospitalization numbers were already declining significantly before the onset of the COVID-19 pandemic in both diagnostic groups. For cervical disc herniation (M50.+), the pre-2020 trend corresponded to a decrease of 644 cases per year (β = −644 cases/year; 95% CI −952 to −337; p = 0.002). An additional immediate level decrease of 4118 hospitalizations was observed in 2020 (β = −4118; 95% CI −5728 to −2509; p < 0.001), whereas no significant subsequent change in slope was observed (β = +167 cases/year; 95% CI −485 to +819; p = 0.553). Similarly, hospitalizations for thoracolumbar disc herniation (M51.+) declined by 3849 cases per year before 2020 (β = −3849 cases/year; 95% CI −4867 to −2831; p < 0.001), followed by an additional immediate level decrease of 16,091 hospitalizations in 2020 (β = −16,091; 95% CI −21,422 to −10,761; p < 0.001). The estimated slope became less negative thereafter (slope change: β = +2214 cases/year; 95% CI + 54 to +4373; p = 0.046); however, this slope-change estimate was not robust to HC3 standard-error estimation.

The most affected age group throughout the study period was 45–60 years. A slight male predominance was consistently observed, with males accounting for 51% of M50.+ and 52.5% of M51.+ cases. Since 2018, a gradual demographic shift toward older patients has become apparent. The proportion of individuals aged ≥80 years increased from 2.1% in 2014 to 4.4% in 2023, and the share of patients aged >60 years steadily increased over the same period. Overall, the age distribution differed significantly between patients with cervical and thoracolumbar disc herniation (Pearson's χ^2^ = 1980.1, df = 3, p < 0.0001). (Fig. 3).

**Fig. 3:**
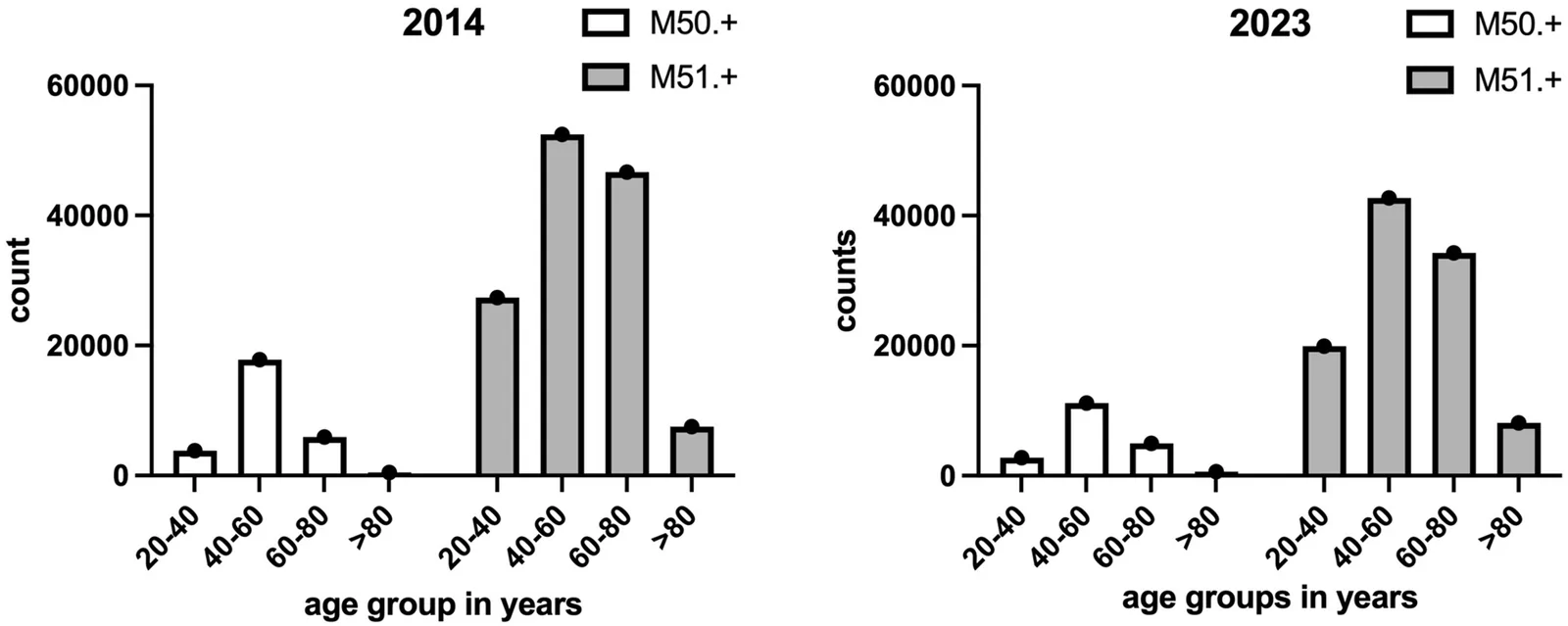
Age distribution of hospitalized patients with cervical (M50.+) and thoracolumbar (M51.+) disc herniation in 2014 and 2023.

Across the entire cohort, 7,129,907 OPS-coded procedures were recorded during inpatient treatment. These values represent cumulative OPS-coded procedure counts rather than numbers or proportions of individual patients undergoing the respective interventions. As multiple procedures may be coded during a single hospitalization, the available aggregated data do not permit calculation of the proportion of hospitalizations involving at least one surgical procedure. Among patients with M51.+ diagnoses, 1,009,202 thoracolumbar sequestrectomies, 589,065 fusion procedures, and 80,722 revision procedures were identified. In addition, 519,434 interventional spinal injections were documented, including 285,401 periradicular therapies and 167,944 facet joint infiltrations (Fig. 4).

**Fig. 4:**
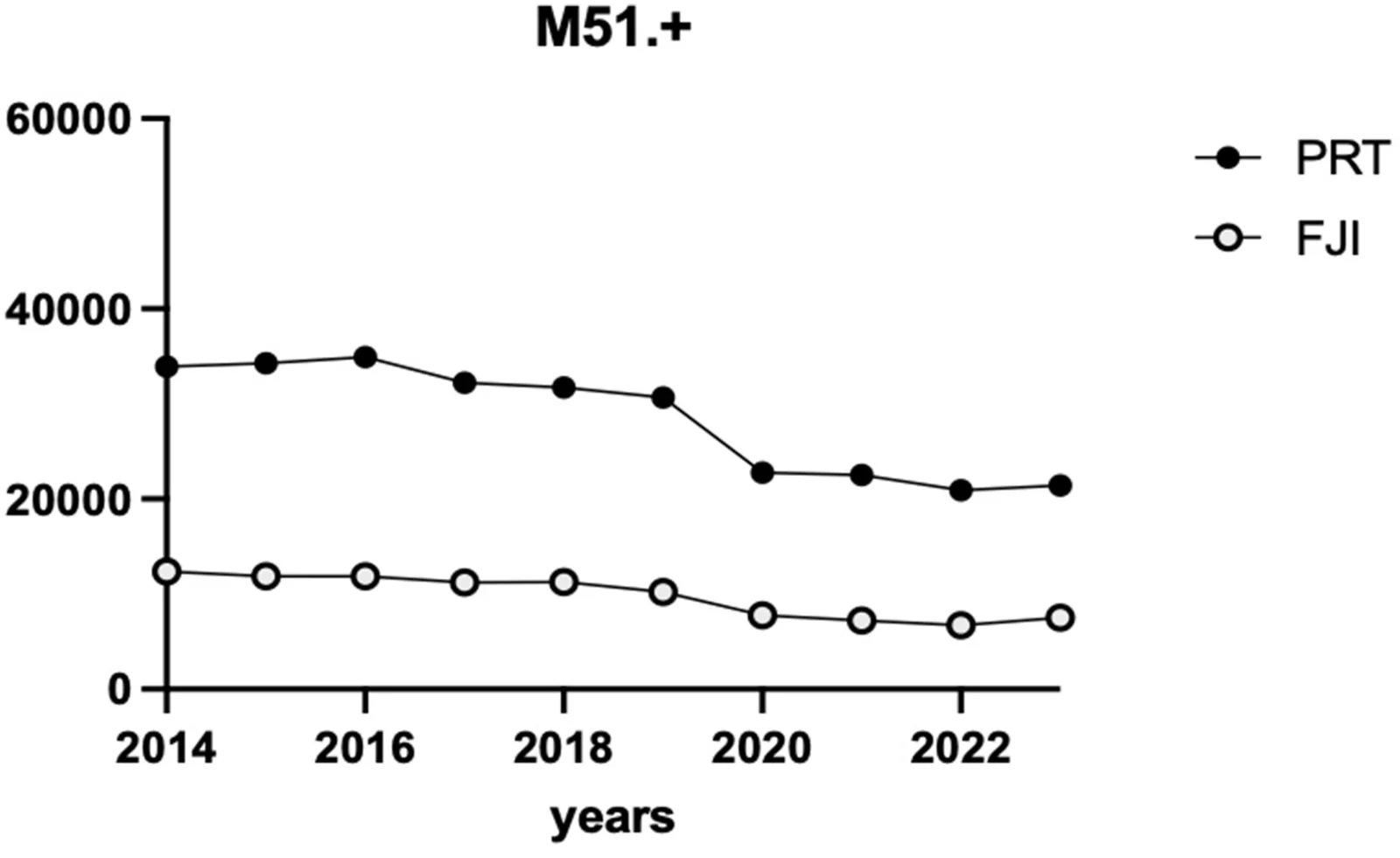
Annual number of inpatient periradicular therapies (PRT) and facet joint infiltrations (FJI) performed in patients with thoracolumbar disc herniation (M51.+) between 2014 and 2023.

Among patients with M50.+ diagnoses, 175,495 cervical discectomies, 115,641 fusion procedures, and 684 revision procedures were identified. Furthermore, 103,451 interventional pain procedures were performed (Fig. 5.)

**Fig. 5:**
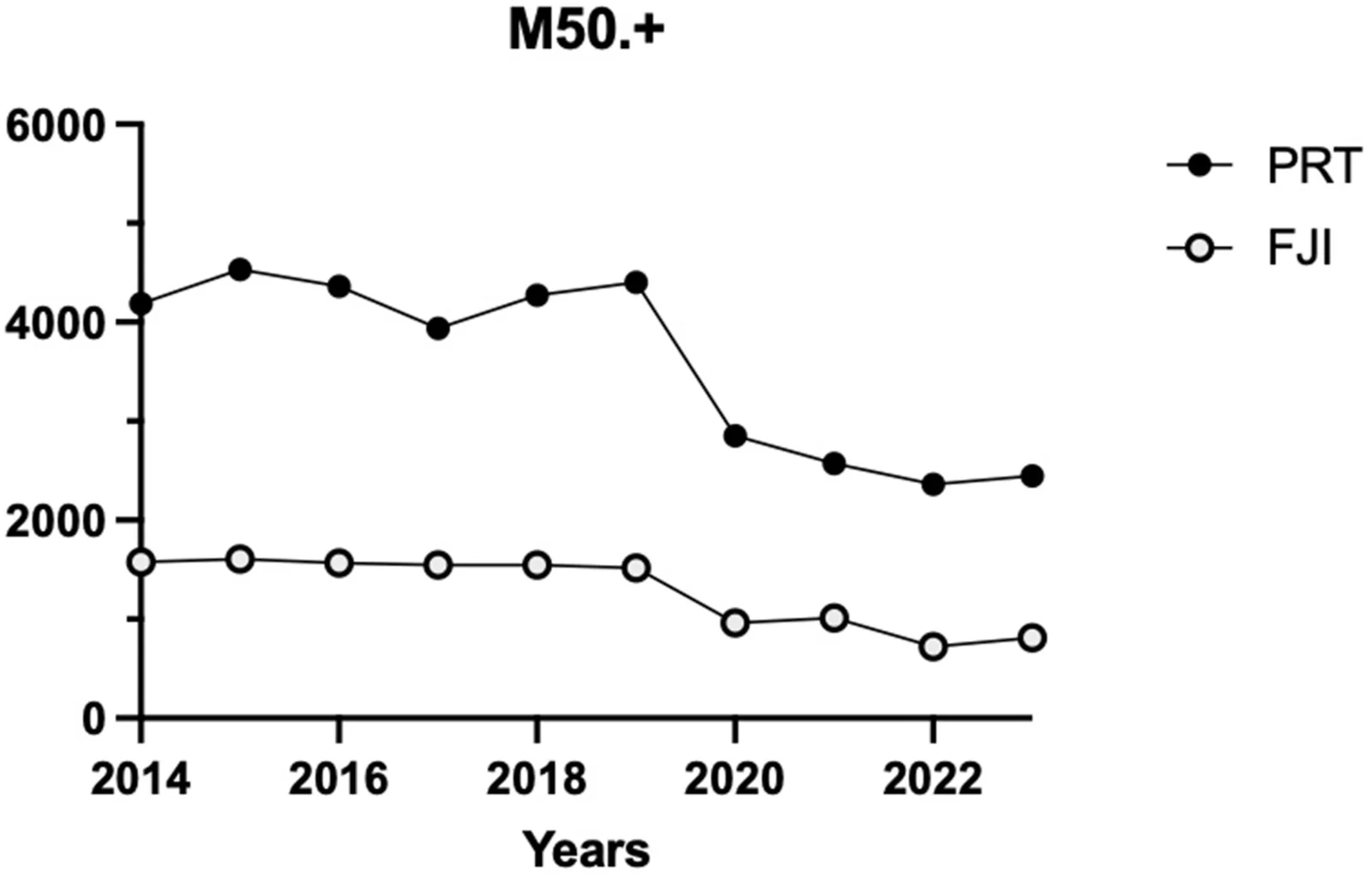
Annual number of inpatient periradicular therapies (PRT) and facet joint infiltrations (FJI) performed in patients with cervical disc herniation (M50.+) between 2014 and 2023.

While operative volumes declined only moderately beginning in 2020, interventional procedures showed a substantially steeper decline. Linear regression demonstrated significant reductions for both periradicular therapies (β = −260.3 procedures/year; 95% CI −384.0 to −136.7; R^2^ = 0.747; p = 0.0013) and facet joint infiltrations (β = −106.8 procedures/year; 95% CI −150.5 to −63.2; R^2^ = 0.799; p < 0.001). Overall, injection-based treatments declined from 66,577 procedures in 2014 to 37,690 in 2023 (−43%), whereas operative volumes showed only a comparatively modest reduction.

Mean length of stay decreased continuously throughout the study period (Fig. 6).

**Fig. 6:**
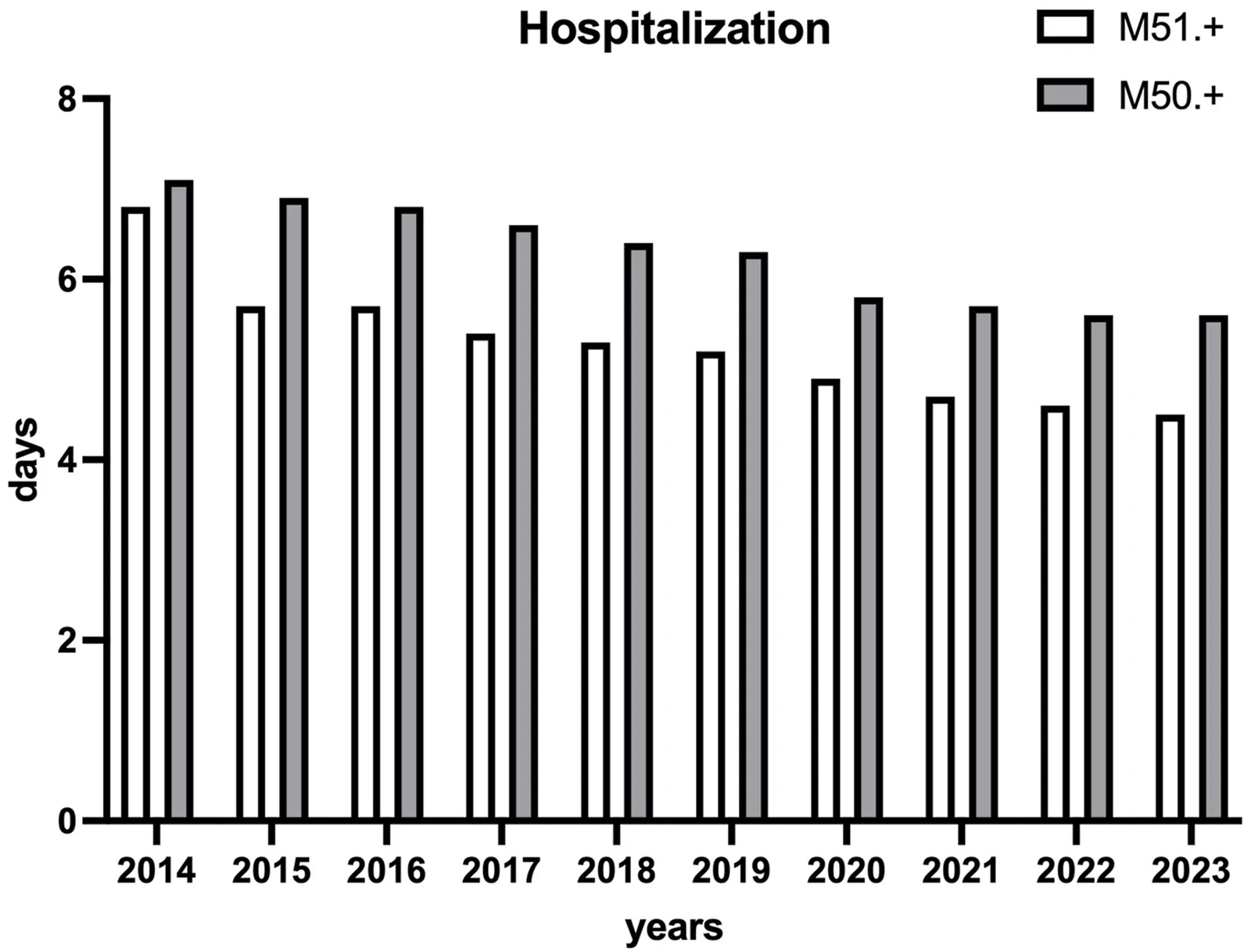
Mean length of hospital stay for cervical (M50.+) and thoracolumbar (M51.+) disc herniation in Germany from 2014 to 2023.

Linear regression demonstrated significant reductions in both diagnostic groups. For cervical disc herniation (M50.+), mean length of stay decreased by 0.19 days per year (β = −0.186 days/year; 95% CI −0.214 to −0.157; R^2^ = 0.966; p < 0.001), corresponding to an overall reduction from 7.1 to 5.2 days between 2014 and 2023. Likewise, mean length of stay for thoracolumbar disc herniation (M51.+) decreased by 0.21 days per year (β = −0.212 days/year; 95% CI −0.277 to −0.147; R^2^ = 0.876; p < 0.001), corresponding to a reduction from 6.0 to 4.5 days over the study period.

The most frequent secondary diagnoses were arterial hypertension (6%), diabetes mellitus (2%), hypercholesterolemia (2%), coronary artery disease (1%), and obesity (1%). These comorbidities remained proportionally stable throughout the study period. Beginning in 2020, COVID-19 emerged as a secondary diagnosis, peaking in 2021 (112,853 cases) and 2022 (109,963 cases), corresponding to approximately 10% of hospitalized patients in those years. In 2023, this proportion declined to approximately 5%.

## 4 Discussion

This nationwide analysis of nearly 1.5 million inpatient cases provides a comprehensive overview of epidemiological trends and treatment patterns for cervical and thoracolumbar disc herniation in Germany over a 10-year period.

Overall, our findings indicate that disc herniation continues to primarily affect individuals of working age, while an increasing proportion of elderly patients is being diagnosed and treated. Over the past decade, treatment patterns have also shifted, with a substantial decline in injection-based therapies, a comparatively modest reduction in surgical procedures, and a consistent shortening of hospital stay. Moreover, the COVID-19 pandemic resulted in a noticeable but temporary decline in hospitalization rates.

Consistent with prior epidemiological studies, the highest hospitalization rates were observed among patients aged 45–60 years (Sivasubramaniam et al., 2015; Pojskic et al., 2024). This age distribution mirrors findings from Scandinavian and UK spine registries, which similarly report a peak in surgical incidence among middle-aged individuals (Grotle et al., 2019). Degenerative disc disease is strongly associated with cumulative biomechanical loading and occupational strain, which may explain the predominance of cases among active workforces (Grotle et al., 2019; Violante et al.). From a public health perspective, this highlights the continued socioeconomic relevance of disc herniation, particularly in industrialized societies with aging but still economically active populations (Sivasubramaniam et al., 2015).

At the same time, our data demonstrate a clear demographic shift. The proportion of patients aged ≥80 years doubled over the study period. This trend parallels broader demographic developments in Europe, where life expectancy has steadily increased over the past decades, as documented by GBD 2021 Europe Life Expectancy Collaborators (GBD, 2021 Europe Life Expectancy Collaborators, 2025).

Improved imaging availability, broader surgical eligibility in elderly patients, and advances in perioperative management likely contribute to the increasing diagnostic and therapeutic engagement of older individuals (Watanabe et al., 2019). The observed rise in elderly patients underscores the need for tailored perioperative risk stratification and geriatric spine care pathways (Sakai et al., 2023).

Regarding treatment patterns, a divergence between operative and interventional strategies became apparent. While surgical volumes declined only moderately, the use of injection-based therapies decreased substantially over the study period (Soin et al., 2025). Several factors may have contributed to this observation. Updated guideline recommendations increasingly advocate a more selective use of epidural and periradicular injections, as evidence supporting sustained long-term benefit remains limited (Manchikanti et al., 2020; Soin et al., 2025; Chou et al). In addition, changes in reimbursement structures and a potential shift of interventional pain management toward the outpatient setting may have contributed to the decline in inpatient procedure coding. However, the present administrative dataset contains no information on guideline adherence, reimbursement mechanisms, or outpatient procedures. These explanations therefore remain hypotheses and cannot be established from the present analysis.

The segmented regression provides further context for the temporal association between the COVID-19 pandemic and inpatient disc herniation care. Importantly, hospitalization numbers were already declining significantly before 2020 in both diagnostic groups, indicating that the overall decrease observed over the study period cannot be attributed to the pandemic alone. Nevertheless, the onset of the pandemic coincided with an additional abrupt reduction in hospitalizations in 2020 beyond the pre-existing downward trend. This finding is consistent with international reports describing reduced elective spine activity during pandemic-related restrictions (Manchikanti et al.; Oshima et al., 2023; Jankovic et al., 2023). Although hospitalization numbers partially recovered from 2022 onward, they did not return to pre-pandemic levels by the end of the observation period. Given the limited number of annual observations and only four observations from 2020 onward, estimates of subsequent changes in slope should be interpreted cautiously. Accordingly, the present data do not allow firm conclusions regarding long-term post-pandemic trends or structural changes in healthcare utilization.

A particularly notable finding is the consistent reduction in mean length of stay. Over the decade, hospital length of stay decreased by approximately 25% for both thoracolumbar and cervical disc herniation. This trend aligns with international data showing shorter hospital stays following spine surgery, attributed to minimally invasive surgical techniques, standardized perioperative protocols, and enhanced recovery after surgery (ERAS) pathways (Angus et al., 2019; Naftalovich et al., 2019). Multiple studies have shown that ERAS-based spine programs can significantly reduce length of stay without increasing complication or readmission rates (Naftalovich et al., 2019; Bansal et al., 2022; Li et al., 2019). The reduction observed in our analysis likely reflects ongoing optimization of inpatient care structures, earlier mobilization, and improved perioperative efficiency within the German healthcare system (Naftalovich et al., 2019).

The comorbidity profile remained relatively stable throughout the study period, with arterial hypertension, diabetes mellitus, hypercholesterolemia, coronary artery disease, and obesity representing the most frequently documented secondary diagnoses. These findings are consistent with the known cardiometabolic risk profile of middle-aged and elderly patients with degenerative spinal disorders. (Battie et al., 2012).^,^ (Parker et al., 2025)

However, the absolute frequencies observed in our cohort were substantially lower than prevalence estimates reported for the general population. This discrepancy is inherent to the use of administrative hospital data. In the German coding system, secondary diagnoses are recorded according to the German Coding Guidelines (Deutsche Kodierrichtlinien, DKR) only when they are clinically relevant to the respective inpatient stay and influence diagnostic or therapeutic management, nursing care, or resource utilization.

Consequently, stable chronic conditions without direct relevance to the hospitalization may remain uncoded. Therefore, the frequencies reported in the present study should be interpreted as documented inpatient secondary diagnoses rather than true population prevalence estimates.

Several limitations must be acknowledged. As an administrative database study, the analysis relies on coding accuracy and lacks detailed clinical variables such as symptom duration, imaging findings, functional outcome measures, complication rates, and differentiation between acute, chronic, first-time, and recurrent disc herniations. Outpatient management was not captured, potentially underestimating the overall burden of conservative and interventional treatment. Furthermore, causality between the observed temporal trends and changes in healthcare delivery cannot be established. An important limitation concerns the aggregation of thoracic and lumbar disc herniations within the M51.+ category. Thoracic disc herniations are substantially less common than lumbar disc herniations and represent a clinically and epidemiologically distinct entity. However, the aggregated Destatis data available for the present analysis did not permit reliable separation of thoracic and lumbar cases across the entire study period. Consequently, the reported M51.+ trends are predominantly driven by the substantially more frequent lumbar disc herniations and should not be interpreted as providing thoracic-specific epidemiological or treatment estimates. This limits the clinical granularity of our findings, particularly with regard to thoracic disc disease. The administrative dataset further provides aggregated OPS procedure counts rather than patient-level treatment information. Multiple OPS codes may be recorded during a single hospitalization, while individual patients cannot be tracked across separate admissions. Consequently, the proportion of hospitalizations involving at least one surgical procedure, the number of individual patients undergoing surgery, and repeated interventions at the patient level could not be determined. Patient-level risk adjustment was likewise not feasible. The segmented regression was based on only ten annual observations, including four observations from 2020 onward, limiting statistical power for detecting changes in post-2020 slopes. Although residual diagnostics and sensitivity analyses were performed, estimates of slope changes should therefore be interpreted cautiously. In addition, multiple prespecified epidemiological and treatment-related outcomes were evaluated without formal adjustment for multiple testing. Accordingly, the possibility of type I error should be considered when interpreting individual p-values. Finally, the findings should be interpreted within the context of the German healthcare system. Differences in reimbursement structures, coding practices, healthcare organization, and thresholds for hospitalization may limit direct transferability of these results to other healthcare systems.

Nevertheless, nationwide coverage and a large sample size provide a robust real-world overview of inpatient disc herniation care across an entire healthcare system.

In conclusion, disc herniation in Germany remains predominantly a condition of the working-age population but is increasingly diagnosed and treated in elderly patients. Inpatient hospitalizations declined substantially over the study period, with segmented regression demonstrating that this decline preceded the COVID-19 pandemic and was accompanied by an additional abrupt reduction in 2020. Injection-based therapies declined substantially, whereas operative volumes showed only a moderate reduction, although the administrative data do not permit patient-level treatment estimates. Mean length of stay decreased by approximately one quarter over the past decade. Together, these findings provide nationwide benchmarking data on demographic and treatment-related changes in inpatient spine care in Germany while highlighting important limitations inherent to aggregated administrative data.

## Declaration of competing interests

The authors declare that they have no known competing financial interests or personal relationships that could have appeared to influence the work reported in this paper.
